# Supplementary material for: Complexome profiling of the Chlamydomonas psb28 mutant reveals TEF5 as an early PSII assembly factor
Source: Plant Cell. 2025 Mar 17;37(6):koaf055. doi: 10.1093/plcell/koaf055 (PMC12164590; doi:10.1093/plcell/koaf055)
Supplement: koaf055_Supplementary_Data [file koaf055_supplementary_data.zip › psb28_interactive.zip!/plots/intensity/qualityPlots/peptides/_Cre09.g394436.t1.1.html]

### Cre09.g394436.t1.1

Trivial name:   
SeqCoverage [median]: 24.50  
Mass: 79.65 kDa  
  
 

| Sequence | MissedCleavage | Mass [Da] | wtIntensity | muIntensity | Score |
| --- | --- | --- | --- | --- | --- |
| AADVGADLVGK | 0 | 1014.53 | 60309.90 | 147287.05 | 208.60 |
| AADVGADLVGKVEK | 1 | 1370.74 | 93591.23 | 331132.38 | 174.24 |
| AADVGADLVGKVEKDIPEDDPR | 2 | 2308.15 | 0.00 | 3299.44 | 44.50 |
| AALAMVHEVR | 0 | 1095.59 | 37545.73 | 71257.93 | 104.39 |
| AALAMVHEVRR | 1 | 1251.69 | 25154.23 | 80995.67 | 140.49 |
| AAVIGDTVGDPLK | 0 | 1254.68 | 7427.31 | 88496.65 | 161.67 |
| AAVIGDTVGDPLKDTSGPSLNILIK | 1 | 2493.37 | 2265.00 | 0.00 | 80.84 |
| AKIDMIHSSILDPR | 1 | 1594.85 | 855.27 | 28966.64 | 135.47 |
| DIPEDDPR | 0 | 955.42 | 6529.65 | 22435.68 | 86.83 |
| DTSGPSLNILIK | 0 | 1256.70 | 3587.63 | 30915.60 | 155.15 |
| ERTDALDAAGNTTAAIGK | 1 | 1773.89 | 7386.92 | 26934.20 | 128.11 |
| EYLLEEEQR | 0 | 1207.57 | 7786.05 | 30787.52 | 221.70 |
| EYLLEEEQRGGEEEIVAK | 1 | 2120.03 | 3331.69 | 176696.44 | 157.11 |
| GGEEEIVAK | 0 | 930.47 | 25135.10 | 56900.04 | 215.39 |
| IDMIHSSILDPR | 0 | 1395.72 | 14135.78 | 97409.11 | 166.04 |
| KYIEAGATEHAR | 1 | 1344.68 | 40313.74 | 124910.93 | 229.40 |
| QFNTIAGLMEGTAR | 0 | 1507.75 | 0.00 | 4434.53 | 151.26 |
| QFNTIAGLMEGTARPDYK | 1 | 2010.98 | 0.00 | 8423.66 | 113.28 |
| QFNTIAGLMEGTARPDYKR | 2 | 2167.08 | 0.00 | 13171.52 | 144.50 |
| RQFNTIAGLMEGTAR | 1 | 1663.85 | 0.00 | 1132.50 | 71.98 |
| RQFNTIAGLMEGTARPDYK | 2 | 2167.08 | 0.00 | 702.64 | 73.29 |
| RQFNTIAGLMEGTARPDYKR | 3 | 2323.19 | 0.00 | 885.39 | 59.51 |
| RVAAIQMTGGR | 1 | 1158.63 | 0.00 | 3601.50 | 88.06 |
| SVGKAALAMVHEVRR | 2 | 1622.90 | 0.00 | 2538.33 | 70.98 |
| TDALDAAGNTTAAIGK | 0 | 1488.74 | 111965.92 | 279596.51 | 359.65 |
| TSADELR | 0 | 790.38 | 33646.70 | 98157.09 | 224.39 |
| VAAIQMTGGR | 0 | 1002.53 | 20111.69 | 73295.29 | 177.91 |
| VEKDIPEDDPR | 1 | 1311.63 | 1020.05 | 3594.23 | 90.73 |
| VGGGIYTK | 0 | 793.43 | 130120.04 | 211867.99 | 157.21 |
| VIAEIEHTLK | 0 | 1151.65 | 23992.94 | 161323.07 | 135.86 |
| VLSSQNGR | 0 | 859.45 | 11383.09 | 28966.62 | 151.56 |
| VLSSQNGREYLLEEEQR | 1 | 2049.01 | 0.00 | 597.85 | 83.37 |
| VLSSQNGREYLLEEEQRGGEEEIVAK | 2 | 2961.47 | 4435.60 | 17774.81 | 39.88 |
| YIEAGATEHAR | 0 | 1216.58 | 144723.72 | 321076.66 | 166.29 |
|  |  |  | 816755.0 | 2549565.5 |  |
